# 4-((*E*)-{2-[*N*-(1,5-Dimethyl-3-oxo-2-phenyl-2,3-dihydro-1*H*-pyrazol-4-yl)carboximido­yl]benzyl­idene}amino)-1,5-dimethyl-2-phenyl-2,3-dihydro-1*H*-pyrazol-3-one

**DOI:** 10.1107/S1600536811039158

**Published:** 2011-09-30

**Authors:** Kim Potgieter, Eric Hosten, Thomas Gerber, Richard Betz

**Affiliations:** a Nelson Mandela Metropolitan University, Summerstrand Campus, Department of Chemistry, University Way, Summerstrand, PO Box 77000, Port Elizabeth, 6031, South Africa

## Abstract

The title compound, C_30_H_28_N_6_O_2_, is a symmetric diimine derived from *ortho*-dibenzaldehyde. Both C=N bonds are (*E*)-configured. The terminal *N*-bonded phenyl groups adopt staggered conformations relative to their respective parent heterocycles, the relevant least-squares planes inter­sect at angles of 32.35 (11) and 38.59 (10)°. In the crystal, C—H⋯O contacts connect the mol­ecules into chains along the *b* axis and give rise to a *C* ^1^_1_(14)*C* ^1^_1_(14) and a *R* ^2^_2_(12) pattern on different levels of graph-set analysis. The shortest inter­centroid distance between two centroids was found at 4.2074 (11) Å between the two five-membered heterocycles.

## Related literature

For the crystal structure of another diimine capable of acting as a chelate ligand, see: Yumata *et al.* (2011 ▶). For graph-set analysis of hydrogen bonds, see: Etter *et al.* (1990 ▶); Bernstein *et al.* (1995 ▶). For details on puckering analysis, see: Cremer & Pople (1975 ▶). For general information about the chelate effect, see: Gade (1998 ▶).
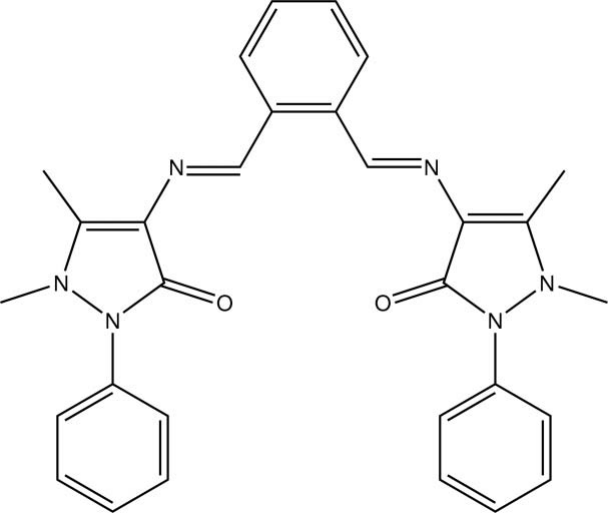


## Experimental

### 

#### Crystal data

C_30_H_28_N_6_O_2_
*M* *_r_* = 504.58
Monoclinic, 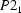

*a* = 12.6048 (2) Å
*b* = 7.3389 (2) Å
*c* = 14.3877 (3) Å
β = 107.622 (1)°
*V* = 1268.48 (5) Å^3^
*Z* = 2
Mo *K*α radiation
μ = 0.09 mm^−1^
*T* = 200 K
0.33 × 0.15 × 0.08 mm

#### Data collection

Bruker APEXII CCD diffractometer
12317 measured reflections
3399 independent reflections
2806 reflections with *I* > 2σ(*I*)
*R*_int_ = 0.032

#### Refinement

*R*[*F* ^2^ > 2σ(*F* ^2^)] = 0.036
*wR*(*F* ^2^) = 0.079
*S* = 1.01
3399 reflections
347 parameters
1 restraint
H-atom parameters constrained
Δρ_max_ = 0.15 e Å^−3^
Δρ_min_ = −0.18 e Å^−3^

### 

Data collection: *APEX2* (Bruker, 2010 ▶); cell refinement: *SAINT* (Bruker, 2010 ▶); data reduction: *SAINT*; program(s) used to solve structure: *SHELXS97* (Sheldrick, 2008 ▶); program(s) used to refine structure: *SHELXL97* (Sheldrick, 2008 ▶); molecular graphics: *ORTEPIII* (Farrugia, 1997 ▶) and *Mercury* (Macrae *et al.*, 2008 ▶); software used to prepare material for publication: *SHELXL97* and *PLATON* (Spek, 2009 ▶).

## Supplementary Material

Crystal structure: contains datablock(s) I, global. DOI: 10.1107/S1600536811039158/bh2383sup1.cif

Supplementary material file. DOI: 10.1107/S1600536811039158/bh2383Isup2.cdx

Structure factors: contains datablock(s) I. DOI: 10.1107/S1600536811039158/bh2383Isup3.hkl

Additional supplementary materials:  crystallographic information; 3D view; checkCIF report

## Figures and Tables

**Table 1:** Hydrogen-bond geometry (Å, °)

*D*—H⋯*A*	*D*—H	H⋯*A*	*D*⋯*A*	*D*—H⋯*A*
C45—H45*A*⋯O2^i^	0.98	2.59	3.535 (2)	161
C55—H55*A*⋯O1^ii^	0.98	2.61	3.536 (3)	158

Symmetry codes: (i) 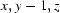; (ii) 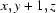.

**Fig. 1.**
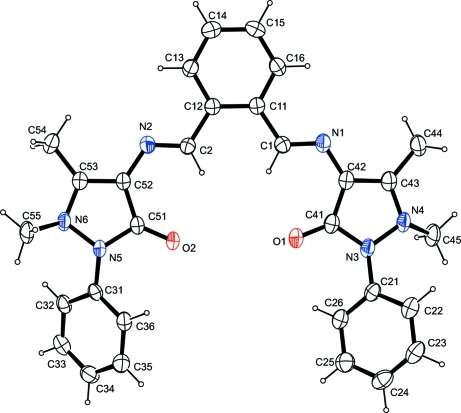
The molecular structure of the title compound, with atom labels and anisotropic displacement ellipsoids (drawn at 50% probability level).

**Fig. 2.**
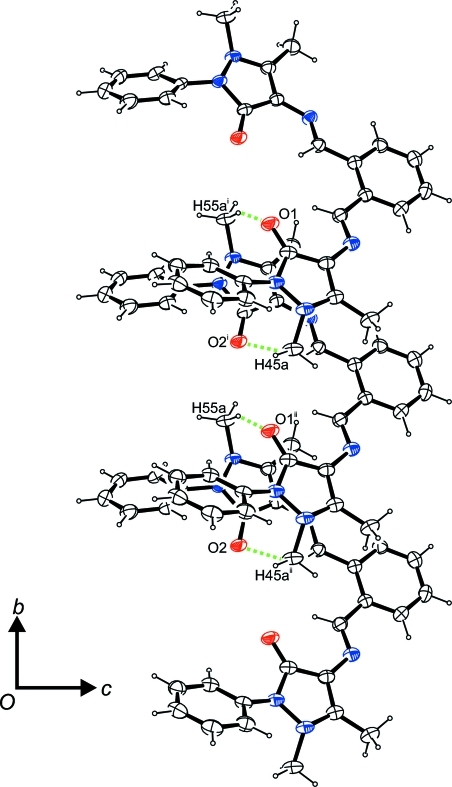
Intermolecular contacts, viewed along [0 - 1 0]. Symmetry operators: ix, y - 1, z; iix, y + 1, z.

**Fig. 3.**
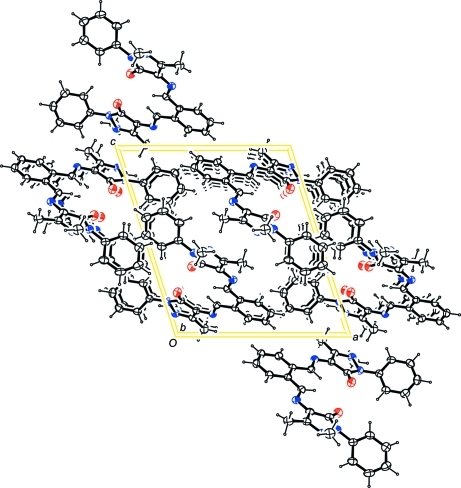
Molecular packing of the title compound, viewed along [0 1 0] (anisotropic displacement ellipsoids drawn at 50% probability level).

## supplementary crystallographic information

### Comment

Chelate ligands have found widespread use in coordination chemistry due to the enhanced thermodynamic stability of resultant coordination compounds in relation to metal complexes exclusively applying comparable monodentate ligands (Gade, 1998). In our continuous efforts in elucidating the rules guiding the formation of coordination compounds applying nitrogen-containing chelate ligands, we determined the structure of the title compound to allow for comparative studies in envisioned coordination compounds. Structural information about another diimine capable of acting as a chelate ligand is apparent in the literature (Yumata *et al.*, 2011).

Both C=N double bonds are (*E*)-configured. The least-squares planes defined by the five-membered heterocycles on the one hand and the central phenyl moiety on the other hand enclose angles of 3.16 (10) and 4.47 (10)°, respectively. The nitrogen-bonded phenyl moieties adopt staggered conformations relative to their respective parent heterocycles, the relevant least-squares planes intersect at angles of 32.35 (11) and 38.59 (10)°. A conformation analysis of the five-membered heterocycles (Cremer & Pople, 1975) is invariably precluded by the small puckering amplitude (Fig. 1).

In the crystal, C–H···O contacts whose range falls by more than 0.1 Å below the sum of van-der-Waals radii are present. These are observed between H atoms of the methyl groups and the ketonic O atoms. In terms of graph-set analysis (Etter *et al.*, 1990; Bernstein *et al.*, 1995), the descriptor for these interactions is *C*^1^_1_(14)*C*^1^_1_(14) on the unitary level and emphasizes the presence of two antidromic chains whereas a *R*^2^_2_(12) descriptor on the binary level highlights the existence of cyclic patterns. In total, the molecules are connected to infinite chains along the crystallographic *b* axis. The shortest intercentroid distance between two centers of gravity was found at 4.2074 (11) Å (Fig. 2).

The packing of the title compound in the crystal is shown in Figure 3.

### Experimental

A solution of 0.99 g of phthalaldehyde in 20 cm^3^ of methanol was added dropwise to a stirred solution of 3.00 g of 4-aminoantipyrine in 30 cm^3^ of methanol. The solution was refluxed under nitrogen for 15 minutes. Upon cooling, a yellow precipitate formed which was filtered and dried under reduced pressure. The product was recrystallized from methanol to produce yellow crystals.

### Refinement

Aromatic carbon-bound H atoms were placed in calculated positions (C—H 0.95 Å) and were included in the refinement in the riding model approximation, with *U*(H) set to 1.2*U*_eq_(C). The H atoms of the methyl groups (C—H 0.98 Å) were allowed to rotate with a fixed angle around the C—C bond to best fit the experimental electron density [HFIX 137 in the *SHELX* program suite (Sheldrick, 2008)], with *U*(H) set to 1.5*U*_eq_(C).

### Crystal data

**Table:**

C_30_H_28_N_6_O_2_	*F*(000) = 532
*M**_r_* = 504.58	*D*_x_ = 1.321 Mg m^−^^3^
Monoclinic, *P*2_1_	Mo *K*α radiation, λ = 0.71073 Å
Hall symbol: P 2yb	Cell parameters from 5101 reflections
*a* = 12.6048 (2) Å	θ = 2.6–28.2°
*b* = 7.3389 (2) Å	µ = 0.09 mm^−^^1^
*c* = 14.3877 (3) Å	*T* = 200 K
β = 107.622 (1)°	Rod, yellow
*V* = 1268.48 (5) Å^3^	0.33 × 0.15 × 0.08 mm
*Z* = 2	

### Data collection

**Table:**

Bruker APEXII CCD diffractometer	2806 reflections with *I* > 2σ(*I*)
Radiation source: fine-focus sealed tube	*R*_int_ = 0.032
graphite	θ_max_ = 28.3°, θ_min_ = 2.6°
φ and ω scans	*h* = −16→16
12317 measured reflections	*k* = −9→9
3399 independent reflections	*l* = −19→18

### Refinement

**Table:**

Refinement on *F*^2^	Primary atom site location: structure-invariant direct methods
Least-squares matrix: full	Secondary atom site location: difference Fourier map
*R*[*F*^2^ > 2σ(*F*^2^)] = 0.036	Hydrogen site location: inferred from neighbouring sites
*wR*(*F*^2^) = 0.079	H-atom parameters constrained
*S* = 1.01	*w* = 1/[σ^2^(*F*_o_^2^) + (0.0471*P*)^2^] where *P* = (*F*_o_^2^ + 2*F*_c_^2^)/3
3399 reflections	(Δ/σ)_max_ < 0.001
347 parameters	Δρ_max_ = 0.15 e Å^−^^3^
1 restraint	Δρ_min_ = −0.18 e Å^−^^3^
0 constraints	

### Special details

**Table:**

Refinement. Due to the absence of a strong anomalous scatterer, the Flack parameter is meaningless. Thus, Friedel opposites (2450 pairs) have been merged and the item was removed from the CIF.

### Fractional atomic coordinates and isotropic or equivalent isotropic displacement parameters (Å^2^)

**Table:**

	*x*	*y*	*z*	*U*_iso_*/*U*_eq_	
O1	0.22361 (12)	−0.1543 (2)	0.36994 (11)	0.0389 (3)	
O2	0.08533 (11)	0.2814 (2)	0.22952 (10)	0.0368 (3)	
N1	0.43019 (13)	−0.2449 (2)	0.30112 (11)	0.0319 (4)	
N2	0.24983 (12)	0.3960 (2)	0.11404 (11)	0.0307 (4)	
N3	0.26332 (13)	−0.4405 (2)	0.44041 (11)	0.0313 (4)	
N4	0.34923 (13)	−0.5696 (2)	0.45284 (12)	0.0329 (4)	
N5	0.01135 (12)	0.5670 (2)	0.17718 (11)	0.0290 (3)	
N6	0.02704 (13)	0.7000 (2)	0.11152 (11)	0.0307 (4)	
C1	0.38269 (15)	−0.1028 (3)	0.25442 (13)	0.0300 (4)	
H1	0.3122	−0.0659	0.2590	0.036*	
C2	0.28998 (15)	0.2528 (3)	0.16189 (13)	0.0287 (4)	
H2	0.2550	0.2004	0.2054	0.034*	
C11	0.43573 (14)	0.0033 (3)	0.19412 (12)	0.0271 (4)	
C12	0.39117 (14)	0.1687 (3)	0.14939 (12)	0.0268 (4)	
C13	0.44707 (14)	0.2614 (3)	0.09350 (13)	0.0320 (4)	
H13	0.4179	0.3738	0.0638	0.038*	
C14	0.54326 (16)	0.1942 (3)	0.08029 (14)	0.0362 (5)	
H14	0.5793	0.2586	0.0411	0.043*	
C15	0.58734 (15)	0.0316 (3)	0.12471 (14)	0.0353 (5)	
H15	0.6542	−0.0152	0.1165	0.042*	
C16	0.53411 (15)	−0.0616 (3)	0.18057 (13)	0.0331 (5)	
H16	0.5650	−0.1728	0.2106	0.040*	
C21	0.19608 (15)	−0.4426 (3)	0.50392 (12)	0.0291 (4)	
C22	0.23326 (16)	−0.5253 (3)	0.59461 (13)	0.0366 (5)	
H22	0.3059	−0.5760	0.6168	0.044*	
C23	0.16325 (18)	−0.5334 (3)	0.65265 (14)	0.0410 (5)	
H23	0.1877	−0.5922	0.7143	0.049*	
C24	0.05832 (18)	−0.4567 (3)	0.62151 (15)	0.0404 (5)	
H24	0.0104	−0.4637	0.6612	0.048*	
C25	0.02366 (17)	−0.3697 (3)	0.53213 (14)	0.0387 (5)	
H25	−0.0478	−0.3143	0.5112	0.046*	
C26	0.09157 (15)	−0.3625 (3)	0.47301 (14)	0.0331 (4)	
H26	0.0670	−0.3030	0.4115	0.040*	
C31	−0.09471 (14)	0.5517 (3)	0.19288 (12)	0.0266 (4)	
C32	−0.19051 (15)	0.6147 (3)	0.12384 (13)	0.0308 (4)	
H32	−0.1866	0.6711	0.0655	0.037*	
C33	−0.29113 (15)	0.5939 (3)	0.14162 (15)	0.0371 (5)	
H33	−0.3572	0.6365	0.0950	0.044*	
C34	−0.29758 (16)	0.5124 (3)	0.22569 (16)	0.0398 (5)	
H34	−0.3677	0.4997	0.2369	0.048*	
C35	−0.20286 (16)	0.4491 (3)	0.29363 (15)	0.0389 (5)	
H35	−0.2074	0.3923	0.3516	0.047*	
C36	−0.10063 (15)	0.4686 (3)	0.27696 (13)	0.0315 (4)	
H36	−0.0349	0.4248	0.3235	0.038*	
C41	0.27970 (16)	−0.2948 (3)	0.38473 (13)	0.0308 (4)	
C42	0.37715 (15)	−0.3447 (3)	0.35641 (13)	0.0300 (4)	
C43	0.41402 (15)	−0.5094 (3)	0.39719 (13)	0.0312 (4)	
C44	0.51017 (17)	−0.6187 (3)	0.38933 (15)	0.0396 (5)	
H44A	0.4831	−0.7214	0.3449	0.059*	
H44B	0.5521	−0.6650	0.4539	0.059*	
H44C	0.5587	−0.5419	0.3640	0.059*	
C45	0.31200 (19)	−0.7619 (3)	0.44471 (16)	0.0425 (5)	
H45A	0.2540	−0.7801	0.3823	0.064*	
H45B	0.2821	−0.7909	0.4984	0.064*	
H45C	0.3753	−0.8419	0.4480	0.064*	
C51	0.08748 (14)	0.4251 (3)	0.18620 (13)	0.0275 (4)	
C52	0.15879 (14)	0.4847 (3)	0.12943 (12)	0.0271 (4)	
C53	0.12091 (14)	0.6489 (3)	0.08866 (13)	0.0293 (4)	
C54	0.16732 (17)	0.7650 (3)	0.02611 (16)	0.0416 (5)	
H54A	0.1097	0.7886	−0.0358	0.062*	
H54B	0.2302	0.7024	0.0135	0.062*	
H54C	0.1927	0.8808	0.0593	0.062*	
C55	0.01693 (18)	0.8901 (3)	0.14159 (16)	0.0388 (5)	
H55A	0.0748	0.9148	0.2033	0.058*	
H55B	−0.0566	0.9082	0.1502	0.058*	
H55C	0.0259	0.9734	0.0913	0.058*	

### Atomic displacement parameters (Å^2^)

**Table:**

	*U*^11^	*U*^22^	*U*^33^	*U*^12^	*U*^13^	*U*^23^
O1	0.0467 (8)	0.0251 (8)	0.0483 (8)	0.0089 (6)	0.0195 (7)	0.0109 (7)
O2	0.0414 (7)	0.0240 (7)	0.0490 (8)	0.0088 (6)	0.0198 (6)	0.0133 (7)
N1	0.0374 (8)	0.0270 (9)	0.0305 (8)	0.0042 (7)	0.0091 (7)	0.0037 (7)
N2	0.0318 (8)	0.0291 (9)	0.0310 (8)	0.0045 (7)	0.0092 (6)	0.0042 (7)
N3	0.0403 (8)	0.0223 (8)	0.0312 (8)	0.0030 (7)	0.0107 (7)	0.0050 (7)
N4	0.0438 (9)	0.0217 (9)	0.0321 (8)	0.0054 (7)	0.0100 (7)	0.0052 (7)
N5	0.0335 (8)	0.0206 (8)	0.0336 (8)	0.0058 (7)	0.0114 (6)	0.0068 (7)
N6	0.0366 (8)	0.0204 (8)	0.0344 (8)	0.0042 (7)	0.0099 (7)	0.0059 (7)
C1	0.0315 (9)	0.0276 (11)	0.0315 (9)	0.0026 (8)	0.0105 (8)	0.0007 (9)
C2	0.0312 (9)	0.0277 (10)	0.0281 (9)	0.0025 (8)	0.0104 (7)	0.0032 (8)
C11	0.0296 (8)	0.0264 (10)	0.0245 (8)	0.0011 (8)	0.0070 (7)	−0.0017 (8)
C12	0.0282 (8)	0.0273 (10)	0.0239 (8)	0.0002 (8)	0.0066 (7)	−0.0012 (8)
C13	0.0335 (9)	0.0291 (10)	0.0332 (10)	−0.0002 (8)	0.0098 (8)	0.0048 (9)
C14	0.0336 (10)	0.0413 (12)	0.0359 (10)	−0.0052 (9)	0.0139 (8)	0.0003 (10)
C15	0.0284 (9)	0.0426 (13)	0.0364 (10)	0.0035 (9)	0.0122 (8)	−0.0048 (10)
C16	0.0323 (9)	0.0329 (12)	0.0336 (10)	0.0062 (9)	0.0091 (8)	−0.0001 (9)
C21	0.0390 (9)	0.0216 (9)	0.0264 (9)	−0.0042 (8)	0.0096 (7)	−0.0013 (8)
C22	0.0444 (10)	0.0319 (11)	0.0301 (9)	−0.0030 (10)	0.0063 (8)	0.0033 (9)
C23	0.0595 (12)	0.0352 (12)	0.0276 (9)	−0.0059 (11)	0.0123 (9)	0.0027 (9)
C24	0.0546 (12)	0.0329 (12)	0.0384 (11)	−0.0105 (10)	0.0214 (9)	−0.0036 (10)
C25	0.0432 (11)	0.0308 (11)	0.0433 (11)	−0.0034 (10)	0.0150 (9)	−0.0026 (10)
C26	0.0415 (10)	0.0262 (10)	0.0297 (9)	−0.0016 (9)	0.0078 (8)	0.0022 (8)
C31	0.0299 (8)	0.0205 (9)	0.0293 (9)	0.0045 (8)	0.0087 (7)	−0.0040 (8)
C32	0.0386 (10)	0.0265 (10)	0.0260 (9)	0.0090 (9)	0.0081 (8)	−0.0019 (8)
C33	0.0336 (9)	0.0340 (12)	0.0395 (10)	0.0091 (9)	0.0049 (8)	−0.0032 (9)
C34	0.0349 (10)	0.0364 (12)	0.0521 (12)	0.0032 (9)	0.0191 (9)	−0.0012 (10)
C35	0.0481 (11)	0.0319 (11)	0.0404 (11)	0.0060 (10)	0.0189 (9)	0.0049 (10)
C36	0.0360 (9)	0.0258 (10)	0.0310 (9)	0.0048 (8)	0.0076 (8)	0.0023 (8)
C41	0.0381 (10)	0.0243 (10)	0.0278 (9)	−0.0006 (8)	0.0063 (8)	0.0004 (8)
C42	0.0351 (9)	0.0259 (10)	0.0265 (9)	0.0018 (8)	0.0059 (8)	0.0015 (8)
C43	0.0385 (10)	0.0273 (10)	0.0247 (8)	0.0030 (9)	0.0049 (8)	−0.0009 (8)
C44	0.0460 (11)	0.0332 (12)	0.0383 (11)	0.0103 (10)	0.0105 (9)	0.0043 (9)
C45	0.0575 (13)	0.0220 (11)	0.0477 (12)	0.0051 (10)	0.0155 (10)	0.0060 (10)
C51	0.0307 (9)	0.0218 (10)	0.0282 (9)	0.0032 (8)	0.0061 (7)	0.0010 (8)
C52	0.0297 (8)	0.0235 (9)	0.0263 (8)	0.0027 (8)	0.0059 (7)	0.0018 (8)
C53	0.0312 (9)	0.0264 (10)	0.0288 (9)	0.0011 (8)	0.0071 (7)	0.0021 (9)
C54	0.0453 (11)	0.0335 (12)	0.0472 (12)	0.0035 (10)	0.0158 (10)	0.0145 (11)
C55	0.0514 (12)	0.0197 (10)	0.0451 (12)	0.0061 (9)	0.0142 (10)	0.0041 (9)

### Geometric parameters (Å, °)

**Table:**

O1—C41	1.232 (2)	C23—H23	0.9500
O2—C51	1.229 (2)	C24—C25	1.383 (3)
N1—C1	1.286 (3)	C24—H24	0.9500
N1—C42	1.392 (2)	C25—C26	1.378 (3)
N2—C2	1.274 (2)	C25—H25	0.9500
N2—C52	1.394 (2)	C26—H26	0.9500
N3—C41	1.389 (2)	C31—C36	1.377 (3)
N3—N4	1.409 (2)	C31—C32	1.390 (2)
N3—C21	1.422 (2)	C32—C33	1.376 (3)
N4—C43	1.378 (2)	C32—H32	0.9500
N4—C45	1.481 (3)	C33—C34	1.374 (3)
N5—C51	1.395 (2)	C33—H33	0.9500
N5—N6	1.413 (2)	C34—C35	1.375 (3)
N5—C31	1.426 (2)	C34—H34	0.9500
N6—C53	1.373 (2)	C35—C36	1.388 (3)
N6—C55	1.478 (3)	C35—H35	0.9500
C1—C11	1.469 (3)	C36—H36	0.9500
C1—H1	0.9500	C41—C42	1.453 (3)
C2—C12	1.476 (3)	C42—C43	1.362 (3)
C2—H2	0.9500	C43—C44	1.486 (3)
C11—C16	1.396 (2)	C44—H44A	0.9800
C11—C12	1.408 (3)	C44—H44B	0.9800
C12—C13	1.396 (3)	C44—H44C	0.9800
C13—C14	1.374 (3)	C45—H45A	0.9800
C13—H13	0.9500	C45—H45B	0.9800
C14—C15	1.388 (3)	C45—H45C	0.9800
C14—H14	0.9500	C51—C52	1.453 (3)
C15—C16	1.375 (3)	C52—C53	1.362 (3)
C15—H15	0.9500	C53—C54	1.482 (3)
C16—H16	0.9500	C54—H54A	0.9800
C21—C22	1.386 (3)	C54—H54B	0.9800
C21—C26	1.387 (3)	C54—H54C	0.9800
C22—C23	1.388 (3)	C55—H55A	0.9800
C22—H22	0.9500	C55—H55B	0.9800
C23—C24	1.381 (3)	C55—H55C	0.9800
			
C1—N1—C42	119.80 (16)	C32—C31—N5	120.91 (16)
C2—N2—C52	120.71 (16)	C33—C32—C31	118.70 (17)
C41—N3—N4	110.38 (14)	C33—C32—H32	120.7
C41—N3—C21	126.90 (16)	C31—C32—H32	120.7
N4—N3—C21	119.75 (15)	C34—C33—C32	121.04 (18)
C43—N4—N3	106.19 (15)	C34—C33—H33	119.5
C43—N4—C45	119.27 (17)	C32—C33—H33	119.5
N3—N4—C45	114.69 (16)	C33—C34—C35	120.20 (18)
C51—N5—N6	110.25 (14)	C33—C34—H34	119.9
C51—N5—C31	125.29 (16)	C35—C34—H34	119.9
N6—N5—C31	119.20 (14)	C34—C35—C36	119.62 (19)
C53—N6—N5	106.08 (14)	C34—C35—H35	120.2
C53—N6—C55	119.00 (17)	C36—C35—H35	120.2
N5—N6—C55	114.48 (15)	C31—C36—C35	119.86 (17)
N1—C1—C11	121.03 (17)	C31—C36—H36	120.1
N1—C1—H1	119.5	C35—C36—H36	120.1
C11—C1—H1	119.5	O1—C41—N3	124.51 (18)
N2—C2—C12	119.71 (17)	O1—C41—C42	130.71 (19)
N2—C2—H2	120.1	N3—C41—C42	104.68 (16)
C12—C2—H2	120.1	C43—C42—N1	123.54 (18)
C16—C11—C12	118.59 (17)	C43—C42—C41	108.06 (17)
C16—C11—C1	118.93 (17)	N1—C42—C41	128.35 (17)
C12—C11—C1	122.48 (16)	C42—C43—N4	110.47 (17)
C13—C12—C11	118.83 (17)	C42—C43—C44	128.76 (19)
C13—C12—C2	118.15 (17)	N4—C43—C44	120.76 (18)
C11—C12—C2	122.98 (17)	C43—C44—H44A	109.5
C14—C13—C12	121.67 (19)	C43—C44—H44B	109.5
C14—C13—H13	119.2	H44A—C44—H44B	109.5
C12—C13—H13	119.2	C43—C44—H44C	109.5
C13—C14—C15	119.46 (19)	H44A—C44—H44C	109.5
C13—C14—H14	120.3	H44B—C44—H44C	109.5
C15—C14—H14	120.3	N4—C45—H45A	109.5
C16—C15—C14	119.93 (18)	N4—C45—H45B	109.5
C16—C15—H15	120.0	H45A—C45—H45B	109.5
C14—C15—H15	120.0	N4—C45—H45C	109.5
C15—C16—C11	121.53 (19)	H45A—C45—H45C	109.5
C15—C16—H16	119.2	H45B—C45—H45C	109.5
C11—C16—H16	119.2	O2—C51—N5	124.56 (17)
C22—C21—C26	120.34 (18)	O2—C51—C52	130.96 (17)
C22—C21—N3	120.80 (17)	N5—C51—C52	104.37 (16)
C26—C21—N3	118.84 (16)	C53—C52—N2	122.88 (17)
C21—C22—C23	119.34 (19)	C53—C52—C51	108.21 (16)
C21—C22—H22	120.3	N2—C52—C51	128.89 (17)
C23—C22—H22	120.3	C52—C53—N6	110.71 (17)
C24—C23—C22	120.54 (19)	C52—C53—C54	128.22 (18)
C24—C23—H23	119.7	N6—C53—C54	121.06 (17)
C22—C23—H23	119.7	C53—C54—H54A	109.5
C23—C24—C25	119.46 (19)	C53—C54—H54B	109.5
C23—C24—H24	120.3	H54A—C54—H54B	109.5
C25—C24—H24	120.3	C53—C54—H54C	109.5
C26—C25—C24	120.7 (2)	H54A—C54—H54C	109.5
C26—C25—H25	119.6	H54B—C54—H54C	109.5
C24—C25—H25	119.6	N6—C55—H55A	109.5
C25—C26—C21	119.52 (18)	N6—C55—H55B	109.5
C25—C26—H26	120.2	H55A—C55—H55B	109.5
C21—C26—H26	120.2	N6—C55—H55C	109.5
C36—C31—C32	120.58 (17)	H55A—C55—H55C	109.5
C36—C31—N5	118.48 (15)	H55B—C55—H55C	109.5
			
C41—N3—N4—C43	−5.0 (2)	C31—C32—C33—C34	0.0 (3)
C21—N3—N4—C43	−166.77 (16)	C32—C33—C34—C35	−0.4 (3)
C41—N3—N4—C45	−138.91 (17)	C33—C34—C35—C36	0.3 (3)
C21—N3—N4—C45	59.3 (2)	C32—C31—C36—C35	−0.6 (3)
C51—N5—N6—C53	−6.49 (19)	N5—C31—C36—C35	−178.99 (19)
C31—N5—N6—C53	−162.23 (16)	C34—C35—C36—C31	0.2 (3)
C51—N5—N6—C55	−139.77 (17)	N4—N3—C41—O1	−172.85 (17)
C31—N5—N6—C55	64.5 (2)	C21—N3—C41—O1	−12.7 (3)
C42—N1—C1—C11	−178.92 (16)	N4—N3—C41—C42	3.94 (19)
C52—N2—C2—C12	−175.70 (16)	C21—N3—C41—C42	164.11 (16)
N1—C1—C11—C16	6.0 (3)	C1—N1—C42—C43	170.82 (18)
N1—C1—C11—C12	−173.96 (17)	C1—N1—C42—C41	−12.0 (3)
C16—C11—C12—C13	−0.1 (2)	O1—C41—C42—C43	175.1 (2)
C1—C11—C12—C13	179.78 (17)	N3—C41—C42—C43	−1.43 (19)
C16—C11—C12—C2	−177.71 (17)	O1—C41—C42—N1	−2.4 (3)
C1—C11—C12—C2	2.2 (3)	N3—C41—C42—N1	−178.94 (17)
N2—C2—C12—C13	7.2 (3)	N1—C42—C43—N4	176.02 (17)
N2—C2—C12—C11	−175.19 (17)	C41—C42—C43—N4	−1.6 (2)
C11—C12—C13—C14	0.8 (3)	N1—C42—C43—C44	−2.9 (3)
C2—C12—C13—C14	178.47 (16)	C41—C42—C43—C44	179.46 (18)
C12—C13—C14—C15	−1.0 (3)	N3—N4—C43—C42	4.0 (2)
C13—C14—C15—C16	0.6 (3)	C45—N4—C43—C42	135.41 (18)
C14—C15—C16—C11	0.0 (3)	N3—N4—C43—C44	−176.99 (16)
C12—C11—C16—C15	−0.2 (3)	C45—N4—C43—C44	−45.6 (3)
C1—C11—C16—C15	179.84 (17)	N6—N5—C51—O2	−171.39 (17)
C41—N3—C21—C22	−137.5 (2)	C31—N5—C51—O2	−17.5 (3)
N4—N3—C21—C22	21.0 (3)	N6—N5—C51—C52	5.15 (19)
C41—N3—C21—C26	43.5 (3)	C31—N5—C51—C52	159.09 (16)
N4—N3—C21—C26	−158.03 (17)	C2—N2—C52—C53	172.22 (17)
C26—C21—C22—C23	2.5 (3)	C2—N2—C52—C51	−9.2 (3)
N3—C21—C22—C23	−176.58 (19)	O2—C51—C52—C53	174.31 (19)
C21—C22—C23—C24	−1.3 (3)	N5—C51—C52—C53	−1.92 (19)
C22—C23—C24—C25	−0.7 (3)	O2—C51—C52—N2	−4.4 (3)
C23—C24—C25—C26	1.5 (3)	N5—C51—C52—N2	179.37 (17)
C24—C25—C26—C21	−0.4 (3)	N2—C52—C53—N6	176.71 (16)
C22—C21—C26—C25	−1.6 (3)	C51—C52—C53—N6	−2.1 (2)
N3—C21—C26—C25	177.43 (18)	N2—C52—C53—C54	−2.3 (3)
C51—N5—C31—C36	49.2 (3)	C51—C52—C53—C54	178.90 (19)
N6—N5—C31—C36	−159.00 (17)	N5—N6—C53—C52	5.20 (19)
C51—N5—C31—C32	−129.2 (2)	C55—N6—C53—C52	135.95 (18)
N6—N5—C31—C32	22.7 (3)	N5—N6—C53—C54	−175.71 (17)
C36—C31—C32—C33	0.5 (3)	C55—N6—C53—C54	−45.0 (3)
N5—C31—C32—C33	178.85 (19)		

### Hydrogen-bond geometry (Å, °)

**Table:**

*D*—H···*A*	*D*—H	H···*A*	*D*···*A*	*D*—H···*A*
C45—H45A···O2^i^	0.98	2.59	3.535 (2)	161.
C55—H55A···O1^ii^	0.98	2.61	3.536 (3)	158.

Symmetry codes: (i) *x*, *y*−1, *z*; (ii) *x*, *y*+1, *z*.
